# Pre-operative Localisation of the Parathyroid Glands in Secondary Hyperparathyroidism: A Retrospective Cohort Study

**DOI:** 10.1038/s41598-019-51265-y

**Published:** 2019-10-10

**Authors:** Takahisa Hiramitsu, Toshihide Tomosugi, Manabu Okada, Kenta Futamura, Makoto Tsujita, Norihiko Goto, Shunji Narumi, Yoshihiko Watarai, Yoshihiro Tominaga, Toshihiro Ichimori

**Affiliations:** Nagoya Daini Red Cross Hospital, Department of Transplant and Endocrine Surgery, 466-8650 2-9 Myoken-cho, Showa-ku, Nagoya, Aichi Japan

**Keywords:** Parathyroid glands, Parathyroid diseases

## Abstract

Complete parathyroidectomy (PTx) is essential during total PTx for secondary hyperparathyroidism (SHPT) to prevent recurrent and persistent hyperparathyroidism. Pre-operative imaging evaluations, including computed tomography (CT), ultrasonography (US), and Tc-99m sestamibi (MIBI) scans, are commonly performed. Between June 2009 and January 2016, 291 patients underwent PTx for SHPT after pre-operative evaluations involving CT, US, and MIBI scans, and the diagnostic accuracies of these imaging modalities for identifying the parathyroid glands were evaluated in 177 patients whose intact parathyroid hormone (PTH) levels were <9 pg/mL after the initial PTx. Additional PTx procedures were performed on 7 of 114 patients whose intact PTH levels were >9 ng/mL after PTx, and the diagnostic validities of the imaging modalities for the remnant parathyroid glands were evaluated. A combination of CT, US, and MIBI scans achieved the highest diagnostic accuracy (75%) for locating bilateral upper and lower parathyroid glands before initial PTx. The accuracies of CT, US, and MIBI scans with respect to locating remnant parathyroid glands before additional PTx were 100%, 28.6%, and 100%, respectively. A combination of CT, US, and MIBI scans is useful for initial PTx for SHPT, and CT and MIBI scans are useful imaging modalities for additional PTx procedures.

## Introduction

During end-stage kidney disease, a lack of vitamin D activation in the kidney and phosphorus excretion into the urine causes hypocalcaemia and hyperphosphatemia, resulting in continuous stimulation of the parathyroid glands and causing secondary hyperparathyroidism (SHPT). In SHPT, enlargement (parathyroid dysplasia) of the usual parathyroid glands, i.e. bilateral upper and lower and sometimes the ectopic parathyroid glands, and the supernumerary parathyroid glands is observed. Ectopic parathyroid glands may result from variability or alteration in embryologic development or migration due to the influence of gravity or negative intrathoracic pressure following enlargement^1^. Supernumerary parathyroid glands may develop from accessory parathyroid fragments arising from the pharyngotracheal duct and are microscopic rests^2,3^. The incidences of ectopic and supernumerary parathyroid glands reported in operative series of patients with end-stage kidney disease are 39.3% and 6.5–37%, respectively^3–7^. On the other hand, primary hyperparathyroidism, which is caused by adenoma, hyperplasia, and rarely carcinoma, may show enlargement of the pathological parathyroid glands and usually causes hypercalcemia^8,9^. The common treatment of primary hyperparathyroidism (except for hyperplasia) is the removal of the pathological parathyroid glands. For pre-operative evaluation of primary hyperparathyroidism, diagnostic imaging studies are necessary to identify the pathological parathyroid glands^8,10^. However, in SHPT, removing the entire parathyroid gland is required because remnant parathyroid glands are continuously stimulated and can cause persistent and recurrent SHPT^3,11^. To prevent persistent and recurrent SHPT, pre-operative diagnostic imaging studies are necessary for localizing as many parathyroid glands as possible, including usual, ectopic and supernumerary parathyroid glands. Differences in the purpose of diagnostic imaging studies for primary and secondary hyperparathyroidism may lead to variations in the diagnostic imaging modalities that are employed.

During the initial total parathyroidectomy (PTx) for SHPT, complete PTx, which involves removing the entire parathyroid glands, is necessary to prevent persistent and recurrent SHPT, because PTx such cases can cause recurrent laryngeal nerve injury as a consequence of adhesions^12,13^. To achieve complete PTx, pre-operative imaging studies are useful for locating the parathyroid glands. Computed tomography (CT), ultrasonography (US), and Tc-99m sestamibi (MIBI) scans are often performed; however, the effectiveness of each modality and their combinations have not been investigated comprehensively in relation to initial and subsequent PTx procedures. Although many reports describe the efficacy of these modalities, either total PTx and transcervical thymectomy were not performed on all of the patients, or the pre-operative and post-operative intact parathyroid hormone (PTH) levels and the incidence of persistent and recurrent SHPT were not considered^4,14–16^. This implies that any remnant parathyroid glands that may have been present, but could not be identified during surgery, were ignored. Ectopic and supernumerary parathyroid glands are often overlooked, and these cause persistent and recurrent hyperparathyroidism (HPT)^17–19^. In the present study, the consistency in the localisation of the parathyroid glands, including the bilateral upper and lower, ectopic, and supernumerary parathyroid glands, between the imaging studies and their intra-operative identification was meticulously evaluated in patients whose post-operative intact PTH values had improved after initial PTx. The accuracy of the imaging studies in relation to additional PTx for persistent and recurrent SHPT was also investigated.

## Results

### Participants

**Figure 1:**
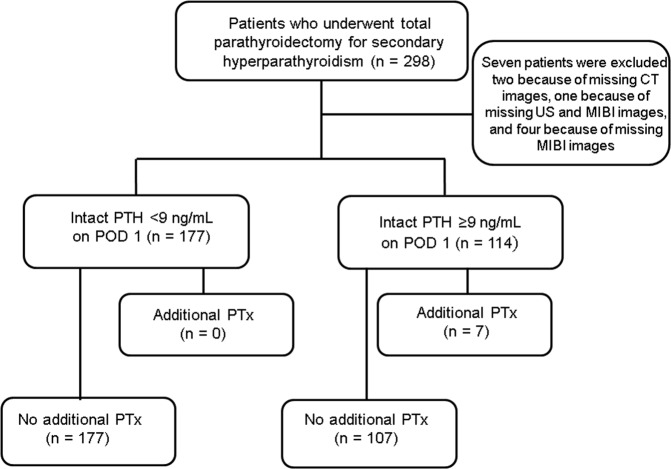
Patient flow chart. CT, computed tomography; US, ultrasonography; MIBI, Tc-99m sestamibi; PTH, parathyroid hormone; POD, post-operative day; PTx, parathyroidectomy.

We monitored 298 patients between June 2009 and January 2016 for a mean observation period of 40.8 (standard deviation [SD] 22.2) months, and none of the patients dropped out of the study during this period. Seven patients were excluded from the study because some of their imaging data were missing. Of the 291 patients who were included in this study, 177 had intact PTH levels of <9 pg/mL, which is the lower limit of a normal intact PTH level, on post-operative day (POD) 1, and 114 had intact PTH levels of >9 pg/mL on POD 1 (Fig. 1).

### Descriptive data

Table 1 summarises the characteristics of the patients who underwent initial PTx stratified according to their intact PTH levels on POD 1. The incidence of additional PTx for persistent and recurrent HPT was significantly lower in patients whose intact PTH levels were <9 pg/mL on POD 1 than in those with levels >9 pg/mL (odds ratio [OR]: 24.77, 95% confidence interval [CI]: 1.400–438.030; *P* = 0.001) (Table 1). Table 2 shows the number and locations of the parathyroid glands, including ectopic and supernumerary parathyroid glands, that were removed from the 177 patients whose intact PTH levels were <9 pg/mL on POD 1. Of the 177 patients, only 5 (2.8%) had 3 parathyroid glands; 39 (22.0%) had 44 supernumerary parathyroid glands (number of parathyroid glands ≥5), comprising 6 left lower, 2 right lower, 1 carotid sheath, and 35 intrathymic glands; and 65 (36.7%) had 90 ectopic parathyroid glands, comprising 2 intrathyroid, 1 mediastinal, and 87 intrathymic glands. Supplementary Fig. S1 shows an MIBI image of a remnant intrathymic parathyroid gland. Table 3 presents the characteristics of the 7 patients who underwent further PTx for persistent and recurrent SHPT. The mean intact PTH level after initial surgery was 157.1 pg/mL, significantly higher than the 9 pg/mL threshold.

**Table 1:** Patient characteristics.

	Intact PTH (POD 1) <9 ng/mL	Intact PTH (POD 1) ≥9 ng/mL	*P* value	Odds ratio	95% CI	
	177	114				
Male sex (n, %)	102 (56.7)	30 (36.1)	**0**.**006**	0.502	0.311	0.811
Mean age (years) (SD)	56.0 (11.0)	55.8 (12.4)	0.732		−2.267	3.222
Mean hemodialysis vintage (months) (SD)	140.3 (83.2)	140.4 (87.1)	0.907			
Mean serum calcium level before initial PTx (mg/dL) (SD)	9.76 (0.91)	9.62 (0.78)	0.163		−0.059	0.349
Mean serum phosphorus level before initial PTx (mg/dL) (SD)	5.67 (1.70)	6.00 (1.57)	0.103		−0.713	0.658
Mean serum intact PTH level before initial PTx (pg/mL) (SD)	591.4 (417.7)	884.9 (708.8)	<**0**.**001**			
Mean serum intact PTH level after initial PTx (pg/mL) (SD)	4.1 (2.0)	62.6 (91.0)	<**0**.**001**			
Mean observation period (months) (SD)	39.5 (21.9)	42.0 (22.1)	0.341		−7.719	2.679
Additional PTx for persistent and recurrent hyperparathyroidism (n [%])	0	7 (6.1)	**0**.**001**	24.77	1.400	438.030

PTH, parathyroid hormone; POD, postoperative day; 95% CI, 95% confidence interval; PTx, parathyroidectomy; SD, standard deviation.

Bold face font denotes statistically significant *P*-values.

**Table 2:** The number and locations of the parathyroid glands.

Number of parathyroid glands	Number of patients	Number of upper and lower parathyroid glands (ectopic parathyroid glands)	Number of supernumerary parathyroid glands
	177	703 (90, comprising 87 intrathymic, 2 intrathyroid, and 1 mediastinal glands)	44, comprising 6 left lower, 2 right lower, 1 carotid sheath, and 35 intrathymic glands
3	5	15 (0)	0
4	133	532 (64, comprising 2 intrathyroid, 1 mediastinal, and 61 intrathymic glands)	0
5	34	136 (22 intrathymic glands)	34, comprising 4 left lower, 2 right lower, and 28 intrathymic glands
6	5	20 (4 intrathymic glands)	10, comprising 1 carotid sheath, 2 left lower, and 7 intrathymic glands

**Table 3:** Characteristics of the patients who underwent additional parathyroidectomies.

	Patient characteristics
	7
Mean duration until additional PTx (months) (SD)	30.7 (17.7)
Mean serum calcium level before additional PTx (mg/dL) (SD)	10.1 (0.8)
Mean serum phosphorus level before additional PTx (mg/dL) (SD)	5.2 (0.8)
Mean serum intact PTH level after initial operation (pg/mL) (SD)	157.1 (106.9)
Mean serum intact PTH level before additional PTx (pg/mL) (SD)	409.0 (183.2)
Mean serum intact PTH level after additional PTx (pg/mL) (SD)	41.0 (37.9)

PTH, parathyroid hormone; PTx, parathyroidectomy; SD, standard deviation.

### Diagnostic validity of each imaging modality alone and of their combinations before initial parathyroidectomy

Table 4 shows the diagnostic validity that was calculated from the consistency of the imaging studies of the typical bilateral upper and lower parathyroid glands in the patients who underwent initial PTx procedures. The accuracies of the imaging modalities used alone were significantly lower than their accuracy when used in combination. A combination of CT, US, and MIBI scanning was associated with the greatest diagnostic accuracy (75.1%), a difference that was significant (*P* < 0.001) (Table 4).

**Table 4:** Comparisons of the diagnoses of the upper and lower parathyroid glands.

Imaging modality	TP	TN	FP	FN	Accuracy	Comparison of accuracy: *P* value, sign test with Bonferroni correction					
						vs. CT	vs. US	vs. MIBI	vs. CT and US	vs. CT and MIBI	vs. US and MIBI
CT	422	3	2	281	0.600	ref	—	—	—	—	—
US	402	4	1	301	0.573	0.999	ref	—	—	—	—
MIBI	299	3	2	404	0.427	**<0.001**	**<0.001**	ref	—	—	—
CT and US	514	3	2	189	0.730	**<0.001**	**<0.001**	**<0.001**	ref	—	—
CT and MIBI scanning	465	3	2	238	0.661	**<0.001**	**<0.001**	**<0.001**	**<0.001**	ref	—
US and MIBI scanning	463	3	2	240	0.658	**<0.001**	**<0.001**	**<0.001**	**<0.001**	0.999	ref
CT, US, and MIBI scanning	529	3	2	174	0.751	**<0.001**	**<0.001**	**<0.001**	**<0.001**	**<0.001**	**<0.001**

TP, True positive; TN, True negative; FP, False positive; FN, False negative; ref, reference; CT, computed tomography; US, ultrasonography; MIBI, Tc—99m sestamibi scan.

Bold face font denotes statistically significant *P*-values.

Table 5 shows the consistency between the imaging modalities and the diagnostic validity in relation to the ectopic parathyroid glands. CT combined with US and CT combined with US and MIBI scanning were significantly more accurate than any of the imaging modalities used alone. The difference in the diagnostic accuracy of CT and US and CT, US, and MIBI scanning was not significant. None of the supernumerary parathyroid glands were identified pre-operatively in this patient series.

**Table 5:** Comparisons of the diagnoses of the ectopic parathyroid glands.

Imaging modality	TP	TN	FP	FN	Accuracy	Comparison of accuracy: P value, sign test with Bonferroni correction					
						vs. CT	vs. US	vs. MIBI	vs. CT and US	vs. CT and MIBI	vs. US and MIBI
CT	43	0	0	47	0.478	ref	—	—	—	—	—
US	33	0	0	57	0.367	0.999	ref	—	—	—	—
MIBI	27	0	0	63	0.300	0.210	0.999	ref	—	—	—
CT and US	54	0	0	36	0.600	**0**.**021**	<**0**.**001**	<**0**.**001**	ref	—	—
CT and MIBI	52	0	0	38	0.578	0.084	**0**.**021**	<**0**.**001**	0.999	ref	—
US and MIBI	44	0	0	46	0.489	0.999	**0**.**021**	<**0**.**001**	0.441	0.999	ref
CT, US, and MIBI	57	0	0	33	0.633	<**0**.**001**	<**0**.**001**	<**0**.**001**	0.999	0.999	<**0**.**001**

TP, True positive; TN, True negative; FP, False positive; FN, False negative; ref, reference; CT, computed tomography; US, ultrasonography; MIBI, Tc—99m sestamibi scan.

Bold face font denotes statistically significant *P*-values.

### Diagnostic validity of each imaging modality in relation to additional parathyroidectomy

Seven parathyroid glands were removed during additional PTx procedures. Two ectopic parathyroid glands were located in the thymus. Two supernumerary parathyroid glands were located in the thymus and mediastinum. These ectopic and supernumerary parathyroid glands were not identified pre-operatively with US. All remnant parathyroid glands were identified with CT and MIBI. The diagnostic accuracies for US were 0% (0/4 glands) for the remnant mediastinal and intrathymic parathyroid glands and 66.7% (2/3 glands) for the typical bilateral upper and lower parathyroid glands (Table 6). The accuracies of CT, US, and MIBI scanning were 100%, 28.6%, and 100%, respectively (Table 7).

**Table 6:** Additional parathyroidectomy results.

Patient	Number of resected parathyroid glands at initial operation	Location of undetected parathyroid gland at initial operation	Location of resected parathyroid gland at additional PTx	CT	US	MIBI
1	3	Right upper	Right upper	Detected	**Undetected**	Detected
2	3	Right upper	Right upper	Detected	Detected	Detected
3	3	Right lower	Intrathymic	Detected	**Undetected**	Detected
4	3	Left lower	Intrathymic	Detected	**Undetected**	Detected
5	4	None	Intrathymic	Detected	**Undetected**	Detected
6	4	None	Mediastinum	Detected	**Undetected**	Detected
7	4	Right lower	Right lower	Detected	Detected	Detected

PTx, parathyroidectomy; CT, computed tomography; US, ultrasonography; MIBI, Tc-99m sestamibi scan.

**Table 7:** Diagnoses during additional parathyroidectomy.

Imaging modality	TP	TN	FP	FN	Accuracy
CT	7	0	0	0	100%
US	2	0	0	5	**28**.**6%**
MIBI	7	0	0	0	100%

TP, true positive; TN, true negative; FP, false positive; FN, false negative; CT, computed tomography; US, ultrasonography; MIBI, Tc-99m sestamibi scan.

US shows significantly lower accuracy compared to the other two modalities.

## Discussion

SHPT is caused by hypocalcaemia and hyperphosphatemia due to the lack of vitamin D activation in the kidney and phosphorus excretion into the urine following kidney failure. Patients with kidney failure are commonly treated with dialysis to adjust their electrolytes. Despite the adjustment, hypocalcaemia and hyperphosphatemia are often observed and treated with a vitamin D, calcium, and phosphate binder. In this study, although the pre-operative mean serum calcium and phosphorus levels were well controlled with these medications, the long haemodialysis vintage (around 140 months in each group) might have caused SHPT^20,21^. Complete PTx, which involves removing the entire parathyroid glands, is necessary for patients with SHPT to prevent persistence and recurrence^10^. However, identifying all of the parathyroid glands is difficult because of their embryology and development of HPT^22^, and in this study, 7 out of 298 patients (2.3%) underwent additional PTx procedures. Considerable variations in the number and locations of the parathyroid glands cause difficulties in their identification^6,22^. Of the 177 patients who underwent the successful removal of all their parathyroid glands and did not need additional PTx, only 5 (2.8%) had 3 parathyroid glands, 39 (22.0%) had supernumerary parathyroid glands, and 65 (36.7%) had 90 ectopic parathyroid glands. The percentages of the patients with ectopic (39.3%) and supernumerary (6.5–37%) parathyroid glands were not lower than those reported previously^4–7^, which indicates that precise PTx procedures were performed in the 177 patients. Undertaking imaging to identify the parathyroid glands before PTx is helpful, because it prevents unsuccessful PTx and unnecessary dissection of tissues that are fragile due to chronic kidney disease^23^. Some studies have investigated the effectiveness of imaging, but the definitions of the complete removal of the parathyroid glands were not clear. The results may also have overestimated the effectiveness of imaging, because ectopic and supernumerary parathyroid glands were overlooked, and the diagnostic validities associated with imaging ectopic and supernumerary parathyroid glands have been rarely investigated for the same reason^16^. In this study, the complete removal of the parathyroid glands was clearly defined, and the results were corroborated by the lack of additional PTx procedures required for persistent and recurrent SHPT^3^.

All the patients in this study underwent CT, US, and MIBI scanning, and the diagnostic validities of the imaging modalities were determined retrospectively. The accuracy of each modality at identifying the standard bilateral upper and lower parathyroid glands was between 42.7% and 60.0%, which was lower than the accuracies of their different combinations. Hence, using one imaging modality is not beneficial for evaluating the location of parathyroid glands, and in particular, the lower diagnostic accuracy of MIBI scans compared with the diagnostic accuracies of CT and US implies that, rather than using MIBI scanning as the main diagnostic imaging modality for pre-operative evaluations of primary HPT, it should be used as an auxiliary imaging modality^24^. Regarding the assessment of the combinations of imaging modalities, the combination of CT, US, and MIBI scanning showed a significantly higher accuracy than other combinations. This implies that a combination of CT, US, and MIBI scanning is the most beneficial for pre-operative localisation of the upper and lower parathyroid glands.

The diagnostic validities of the imaging modalities used to identify the ectopic parathyroid glands were similar to those of the imaging modalities used to identify the upper and lower parathyroid glands. The mediastinal parathyroid glands were successfully identified pre-operatively and removed from 1 of the 177 patients during the initial PTx procedure. The diagnostic accuracies of CT and US and CT, US, and MIBI scanning for ectopic parathyroid gland identification were higher than those associated with any of the imaging modalities used alone or any of the other imaging modality combinations. However, there was no difference in diagnostic accuracy between CT and US and CT, US, and MIBI scanning.

None of the supernumerary parathyroid glands were identified pre-operatively, and 44 supernumerary parathyroid glands were removed. Nine out of 44 supernumerary parathyroid glands were identified during the procedures. The remaining 35 supernumerary parathyroid glands were identified microscopically and could not be identified during pre-operative imaging or surgery^11,18^. Since 8 out of 9 supernumerary parathyroid glands were adjacent to the typically located parathyroid glands and 1 supernumerary parathyroid gland was within the carotid sheath, these were overlooked during the pre-operative imaging assessments. Each of the 8 supernumerary parathyroid glands that were adjacent to the typically located parathyroid glands was identified as one parathyroid gland pre-operatively; hence, pre-operative imaging was not useful for identifying supernumerary parathyroid glands.

While it was difficult to identify the supernumerary parathyroid glands, the results from this study regarding the diagnostic validity of imaging for the identification of the typical upper and lower, and ectopic parathyroid glands demonstrated that a combination of CT, US, and MIBI scans was the most appropriate approach for determining the locations of these glands before the initial PTx procedures.

Persistent and recurrent HPT occurs in 5–30% of patients after PTx for SHPT^25–27^. In our study, 7 out of 291 patients (2.4%) underwent additional PTx procedures for persistent and recurrent HPT. This rate is lower than those reported previously because all the patients underwent total PTx and transcervical thymectomy;^28^ consequently, 35 supernumerary parathyroid glands that were detected microscopically in the thymus were removed from 177 patients. Although this procedure was effective for initial PTx, patients who required additional PTx were encountered. Determining the locations of the parathyroid glands before additional PTx is necessary. The accuracies of CT and MIBI scans before the additional PTx procedures were 100%, but the accuracy of US was lower than the accuracies of CT and MIBI scanning, which indicates that US is not useful for locating parathyroid glands before additional PTx. US could not identify parathyroid glands in the thymus or mediastinum, and its accuracy at identifying parathyroid glands in the typical upper and lower locations was lower (66.7%) than the accuracies of CT (100%) and MIBI scanning (100%). Hence, CT and MIBI scanning may be useful for locating parathyroid glands before additional PTx procedures, but MIBI scanning cannot detect their locations accurately. CT may be necessary to locate parathyroid glands precisely and to avoid recurrent laryngeal nerve injuries caused by scars and unnecessary dissections. CT and MIBI scanning may be useful to confirm the locations of remnant parathyroid glands^29^. For the initial and additional PTx procedures, both CT and MIBI scanning were clearly useful. SPECT/CT imaging has recently been reported to improve planar MIBI diagnostic performance, especially in SHPT^30^. Furthermore, the efficacy of the newest nuclear medicine procedures (i.e. 11C-choline-PET-CT and 18F-choline-PET-CT) have been reported for the pre-operative diagnosis of parathyroid disease^31,32^. The efficacy of these modalities for pre-operative localisation of parathyroid glands in SHPT remains to be investigated.

One limitation associated with this study is its retrospective design; therefore, future prospective randomised studies of the diagnostic accuracy of pre-operative imaging should be undertaken. In conclusion, CT, US, and MIBI scanning are useful for imaging before initial PTx procedures for SHPT, and CT and MIBI scanning are useful for additional PTx procedures.

## Methods

### Ethical considerations

This study was approved by the Nagoya Daini Red Cross Hospital’s Institutional Review Board, and conducted in accordance with the principles of the Declaration of Helsinki.

### Study design

To investigate the diagnostic accuracies of CT, US, and MIBI scanning used alone and in every combination before initial PTx. Patients with intact PTH values of <9 pg/mL on POD 1 were selected to participate in this study. The locations of the parathyroid glands in these patients had been determined pre-operatively by radiologists using each imaging modality alone and in every combination. Determined locations were compared with the locations of the parathyroid glands determined intra-operatively, and consistency was investigated with respect to the bilateral upper and lower, ectopic, and supernumerary parathyroid glands. The diagnostic validity of each imaging modality was also investigated in the patients who required additional PTx procedures. This retrospective cohort study was conducted according to the STrengthening the Reporting of OBservational studies in Epidemiology (STROBE) guidelines.

### Participants

Between June 2009 and January 2016, 298 consecutive patients underwent PTx for SHPT at our hospital. CT, US, and MIBI scanning were performed pre-operatively. One hundred and seventy-seven patients whose intact PTH values were <9 pg/mL on POD 1 participated in an evaluation of the diagnostic validity of the imaging modalities before the initial PTx, and 7 patients participated in an evaluation of the diagnostic validity of the imaging modalities before an additional PTx. The data were collected retrospectively from the patients’ charts and analysed anonymously. As such, the need for informed consent was waived by the relevant institutional review board.

### Indications for an initial parathyroidectomy

The clinical practice guidelines for the management of SHPT in chronic dialysis patients were used to determine a patient’s indications for an initial PTx^33^. Patients who could not continue with calcimimetic agents because of adverse effects were also indicated for initial PTx.

### Pre-operative evaluations of the parathyroid glands

The locations of the parathyroid glands in all the patients were determined pre-operatively by radiologists using CT, US, and planar MIBI scanning. Different radiologists assessed the CT, US, and planar MIBI scans.

### Surgical procedures and locating the parathyroid glands

PTx and transcervical thymectomy with forearm autograft comprised the initial PTx procedure, which was performed under general anaesthesia in all patients. Four surgeons performed the initial and additional PTx procedures, and they determined the locations of the parathyroid glands during the operations. Pathologists examined frozen and paraffin sections of all the parathyroid glands.

### Indications for additional parathyroidectomies

Additional PTx procedures were indicated for patients who were refractory to medication and for patients whose parathyroid glands were identified pre-operatively in the neck or mediastinum using CT, US, or planar MIBI scans^33^.

### Comparisons of the pre-operative imaging evaluations and the intra-operative localisation of the parathyroid glands

The pre-operative imaging evaluations and the intra-operative localisation of the bilateral upper and lower, ectopic, and supernumerary parathyroid glands were compared in relation to the initial PTx. For the additional PTx, the pre-operative imaging evaluations and the intra-operative localisation of the remnant parathyroid glands were compared.

### Statistical analyses

The continuous variables were presented as the means (SDs) and the categorical variables were presented as the numbers and percentages. The statistical analyses were performed using the independent sample t-test or the Mann-Whitney U test for the continuous data, and the chi-squared test or Fisher’s exact test for the categorical variables. The patients were categorised as true positive, true negative, false positive, and false negative, and the accuracies of the imaging studies were evaluated. The accuracies of the imaging studies undertaken before the initial PTx were compared using the sign test. The Bonferroni correction was used to adjust for multiplicity. A value of *P* < 0.05 was considered statistically significant. The statistical analyses were performed using IBM^®^SPSS^®^ software, for Windows, version 23.0 (IBM Corporation, Armonk, NY, USA).

## Supplementary information

Supplementary Figure S1
