# Supplementary material for: Pre-operative Localisation of the Parathyroid Glands in Secondary Hyperparathyroidism: A Retrospective Cohort Study
Source: Sci Rep. 2019 Oct 10;9:14634. doi: 10.1038/s41598-019-51265-y (PMC6787184; doi:10.1038/s41598-019-51265-y)
Supplement: Supplementary file 1 — Supplementary Figure S1 [file 41598_2019_51265_MOESM1_ESM.pdf]

Pre-operative Localisation of the Parathyroid Glands in Secondary Hyperparathyroidism: A Retrospective Cohort Study

Takahisa Hiramitsu*, Toshihide Tomosugi, Manabu Okada, Kenta Futamura, Makoto Tsujita, Norihiko Goto, Shunji Narumi, Yoshihiko Watarai, Yoshihiro Tominaga, Toshihiro Ichimori

Nagoya Daini Red Cross Hospital

Department of Transplant and Endocrine Surgery

466-8650 2-9 Myoken-cho, Showa-ku, Nagoya, Aichi, Japan.

***Corresponding author and address for reprint requests:**

Takahisa Hiramitsu

Nagoya Daini Red Cross Hospital

Department of Transplant and Endocrine Surgery

466-8650 2-9 Myoken-cho, Showa-ku, Nagoya, Aichi, Japan

Email address: thira@nagoya2.jrc.or.jp

Telephone number: +81-52-832-1121

Fax number: +81-52-832-1130

Supplementary Figure S1.

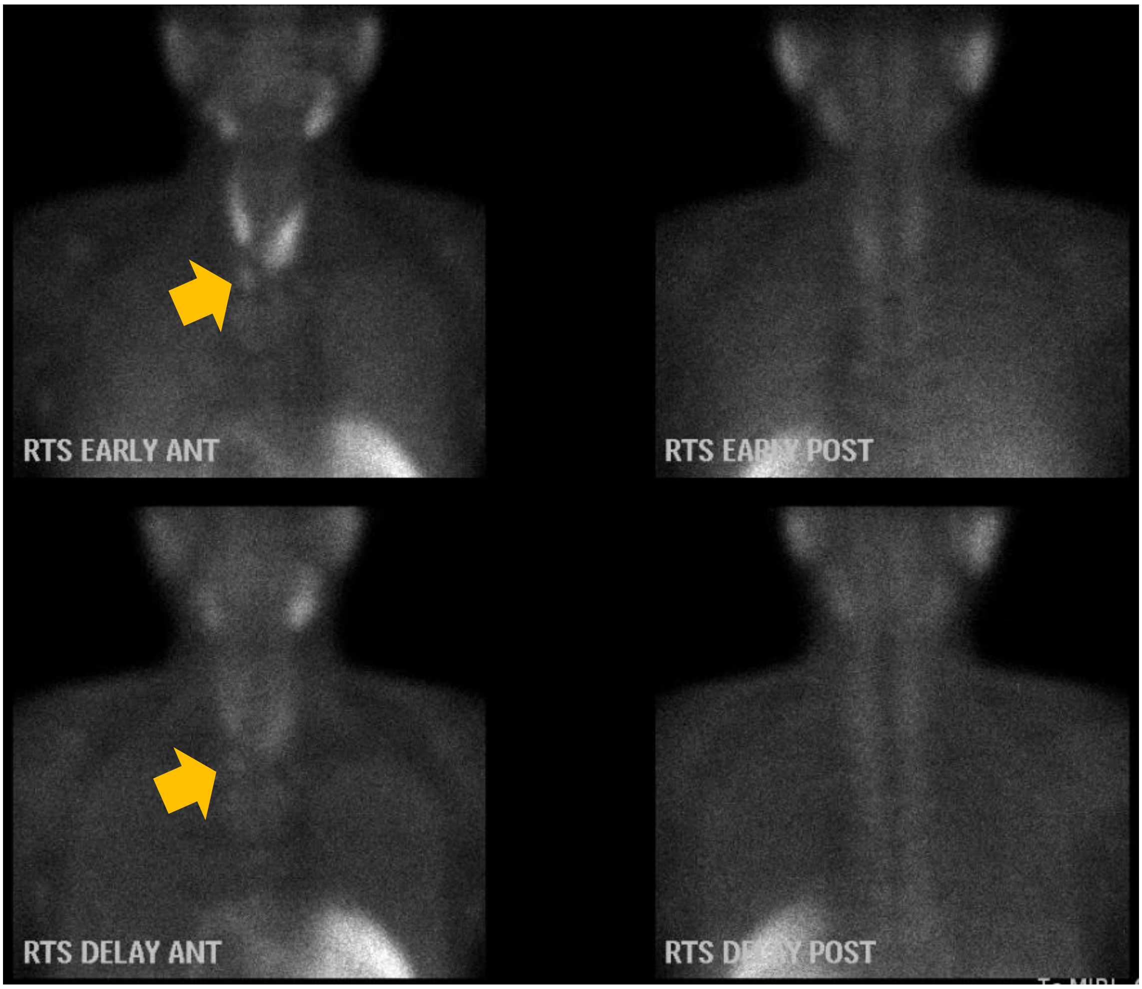

Supplementary Figure S1. MIBI image of a remnant intrathymic parathyroid gland
The yellow arrow shows ectopic parathyroid glands in the thymus.
MIBI, Tc-99m sestamibi
